# Listeria monocytogenes Has Both Cytochrome *bd*-Type and Cytochrome *aa*_3_-Type Terminal Oxidases, Which Allow Growth at Different Oxygen Levels, and Both Are Important in Infection

**DOI:** 10.1128/IAI.00354-17

**Published:** 2017-10-18

**Authors:** David Corbett, Marie Goldrick, Vitor E. Fernandes, Kelly Davidge, Robert K. Poole, Peter W. Andrew, Jennifer Cavet, Ian S. Roberts

**Affiliations:** a School of Biological Sciences, Faculty of Biology Medicine and Health, Manchester Academic Health Science Centre, University of Manchester, Manchester, United Kingdom; b Department of Infection, Immunity and Inflammation, University of Leicester, Leicester, United Kingdom; c Department of Molecular Biology and Biotechnology, University of Sheffield, Sheffield, United Kingdom; University of Illinois at Chicago

**Keywords:** Listeria monocytogenes, cytochromes, foodborne pathogens, host-pathogen interactions, oxygen

## Abstract

Listeria monocytogenes is a foodborne pathogen responsible for a number of life-threatening infections of humans. During an infection, it invades epithelial cells before spreading from the intestine to the cells of the liver and spleen. This requires an ability to adapt to varying oxygen levels. Here, we demonstrate that L. monocytogenes has two terminal oxidases, a cytochrome *bd*-type (CydAB) and a cytochrome *aa*_3_-type menaquinol (QoxAB) oxidase, and that both are used for respiration under different oxygen tensions. Furthermore, we show that possession of both terminal oxidases is important in infection. In air, the CydAB *bd*-type oxidase is essential for aerobic respiration and intracellular replication, and *cydAB* mutants are highly attenuated in mice. In contrast, the QoxAB *aa*_3_-type oxidase is required neither for aerobic respiration in air nor for intracellular growth. However, the *qoxAB* mutants are attenuated in mice, with a delay in the onset of disease signs and with increased survival time, indicating a role for the QoxAB *aa*_3_-type oxidase in the initial stages of infection. Growth of bacteria under defined oxygen conditions revealed that at 1% (vol/vol), both oxidases are functional, and the presence of either is sufficient for aerobic respiration and intracellular replication. However, at 0.2% (vol/vol), both oxidases are necessary for maximum growth. These findings are consistent with the ability of L. monocytogenes to switch between terminal oxidases under different oxygen conditions, providing exquisite adaptation to different conditions encountered within the infected host.

## INTRODUCTION

Listeria monocytogenes is a Gram-positive facultative anaerobe that is responsible for foodborne infections of humans, typified by high mortality rates (1). It is found in a range of environments, including brackish water, soil, and decaying plant material (2, 3). It can grow within a wide temperature range and is resistant to low pH and high salt (4). This versatility may, at least in part, account for the ability of L. monocytogenes to survive food processing and to grow under conditions routinely used for food preservation (5, 6).

In infections following ingestion, L. monocytogenes is able to invade host cells using the InlA and InlB proteins (7) and, after uptake, to escape from the phagosome through the actions of listeriolysin O and the phospholipases PlcA and PlcB (8). Once outside the phagosome, it replicates in the host cell cytosol, polymerizing actin at its pole through the ActA protein to move around the cell and invade neighboring cells (9–12).

Within the environment and the infected host, L. monocytogenes is exposed to varying oxygen tensions that range from fully aerobic to anaerobic conditions. For example, following ingestion of L. monocytogenes in contaminated food, it is exposed to low-oxygen environments in the intestine, while it is exposed to higher levels of oxygen following invasion into host tissue. Under aerobic conditions, the major end products of glucose catabolism are equally lactate, acetate, acetoin, and carbon dioxide (13, 14). In contrast, under anaerobic conditions following fermentation, the major end product is lactate, with small amounts of acetate, formate, ethanol, and carbon dioxide (13). It is unclear what terminal electron acceptors can be used under fermentative anaerobic growth, although fumarate has been suggested to function in this capacity (15). Mutants defective in the *aro* pathway and thus unable to make menaquinone grow anaerobically even under aerobic conditions and are defective for intracellular growth in epithelial cells and attenuated in mice (16). This suggests that intracellular growth of L. monocytogenes and growth *in vivo* are predominantly via aerobic metabolism.

In this paper, we demonstrate that L. monocytogenes expresses two terminal oxidases, a cytochrome *bd*-type (CydAB) and a cytochrome menaquinol *aa*_3_-type (QoxAB) oxidase. Analysis of the growth of mutants defective in either oxidase in different levels of oxygen and during infection demonstrated that the CydAB oxidase is essential for aerobic respiration in air and intracellular replication, whereas the QoxAB oxidase is more important for growth under conditions of low oxygen and is not required for intracellular replication. Crucially, both the Δ*cydAB* and Δ*qoxAB* mutants are highly attenuated in mice, indicating roles for both oxidases during infection. Taken as a whole, these results are consistent with the ability of L. monocytogenes to switch between terminal oxidases under the different oxygen conditions likely to be encountered within the infected host.

## RESULTS

### L. monocytogenes expresses both a cytochrome *bd*-type oxidase and a menaquinol *aa*_3_-type oxidase.

It has previously been reported that L. monocytogenes possesses the genes for two terminal oxidases (17). The *cydABCD* gene cluster encodes the subunits for a cytochrome *bd*-type oxidase, CydAB, along with the CydCD ABC transporter that is essential for the correct membrane insertion of CydAB. In addition, a second gene cluster (*lmo*0013, *lmo*0014, *lmo*0015, and *lmo*0016), here designated *qoxABCD*, encodes the components of a cytochrome *aa*_3_ menaquinol oxidase. This suggested that L. monocytogenes has two terminal oxidases. In other organisms, adaptation to growth in the presence of different oxygen levels and for protection against reactive oxygen molecules has been attributed to the presence of multiple oxidases (18–22). To test the roles of the two oxidases, Δ*cydAB*, Δ*qoxAB*, and Δ*cydAB ΔqoxAB* mutants were generated using the vector pAUL-A (23).

**FIG 1:**
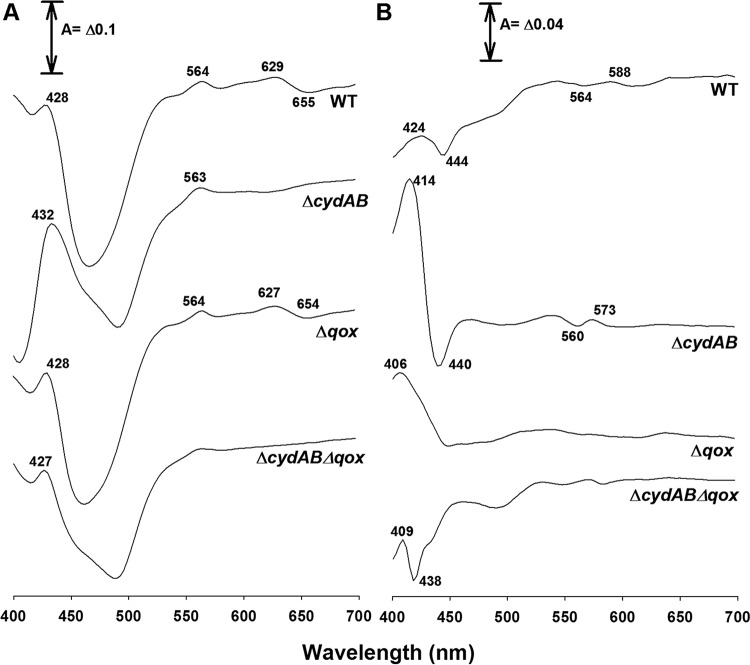
Difference spectra of the strains used in this study. (A) Reduced (dithionite) minus oxidized (persulfate) difference spectra of intact cell suspensions of the indicated strains (WT, wild type). The spectra were recorded at room temperature. (B) Reduced (dithionite) plus CO minus reduced difference spectra of intact cell suspensions of the same strains. The spectra were recorded at room temperature. Distinctive features of the spectra are indicated (nanometers). Other conditions are given in Materials and Methods. Protein concentrations: wild type, 12.97 mg ml^−1^; Δ*cydAB*, 7.57 mg ml^−1^; Δ*qox*, 22.86 mg ml^−1^; Δ*cydABΔqox*, 18.54 mg ml^−1^.

The expression of cytochromes *bd* and *aa*_3_ was confirmed by spectral analysis of both the wild-type EGDe strain and the Δ*cydAB*, Δ*qoxAB*, and Δ*cydAB ΔqoxAB* mutants (Fig. 1). The reduced minus oxidized spectrum (Fig. 1A) of the wild-type strain showed features due to *b*- and/or *c*-type hemes, as well as a clear signature from cytochrome *d* (peak at 629 nm and trough at 655 nm). The CO difference spectrum (Fig. 1B) showed a broad Soret peak centered at 424 nm with multiple contributions and a trough at 444 nm, attributed to hemes *d* and *b*_595_, both components of a *bd*-type oxidase complex. In contrast, the Δ*cydAB* mutant lacked the features beyond 600 nm but showed a broad, prominent trough at 492 nm that was probably due to flavins (Fig. 1A). The CO spectrum (Fig. 1B) again confirmed the lack of CO-binding features of cytochrome *bd* in the red region. The Soret region showed a sharper peak (414 nm) than the wild-type control and a narrow trough (440 nm) that is probably attributable to cytochrome *a*_3_. In contrast, the Δ*qoxAB* mutant gave a reduced minus oxidized spectrum (Fig. 1A) similar to that of the wild type, with clear evidence of cytochrome *bd* at 627 and 654 nm. The CO spectrum (Fig. 1B) showed a minor signal near 640 nm that was probably the CO complex of cytochrome *d* but critically lacked the narrow bands in the Soret attributed to cytochrome *aa*_3_. In summary, the Δ*cydAB* mutant was confirmed to lack cytochrome *bd*. Its absence allowed the characteristics of the additional cytochrome oxidase to be observed, the Soret features of which are consistent with its being a menaquinol *aa*_3_-type oxidase (here designated QoxAB). The lack of these characteristics in the Δ*qoxAB* mutant confirm QoxAB as a cytochrome *aa*_3_-type oxidase.

### The cytochrome *bd* and menaquinol *aa*_3_ oxidases of L. monocytogenes make distinct contributions to growth under different oxygen levels.

To examine the contributions of CydAB and QoxAB to L. monocytogenes aerobic respiration, the growth of the wild type and mutants lacking *cydAB* and/or *qoxAB* was initially examined in air and under anaerobic conditions. During aerobic growth at 37°C in tryptone soya broth (TSB), the Δ*cydAB* mutant had an increased doubling time and reduced growth rate (μ), 0.65 h^−1^, compared to the wild-type strain, 1.10 h^−1^ (Table 1). In contrast, the Δ*qoxAB* mutant had no detectable growth phenotype under these conditions, while the Δ*cydAB ΔqoxAB* double mutant had a growth phenotype similar to that of the Δ*cydAB* single mutant (Table 1). The growth defect in the Δ*cydAB* mutant could be complemented by plasmid pCydAB, in which the *cydAB* genes are cloned in pUNK1 (data not shown). These data are consistent with the cytochrome *bd*, but not the menaquinol *aa*_3_, oxidase being required for aerobic growth in air. Under anaerobic conditions, there was no detectable growth difference between any of the strains (Table 1), demonstrating that neither oxidase is obligatory under these conditions.

**TABLE 1:** Properties of the wild type and Δ*cydAB*, Δ*qoxAB*, and Δ*cydAB* Δ*qoxAB* mutants following growth in TSB

Strain	Aerobic				Anaerobic			
	Td^*a*^ (min)	μ (/h)	Final pH (±SE)	Final *A*_600_ (±SE)	Td (min)	μ (/h)	Final pH (±SE)	Final *A*_600_ (±SE)
WT	37.8	1.10	6.21 ± 0.02	1.669 ± 0.004	96.9	0.43	5.12 ± 0	0.768 ± 0.013
Δ*cydAB*	63.6	0.65	5.28 ± 0.01	1.148 ± 0.024	86.3	0.48	5.12 ± 0	0.771 ± 0.022
Δ*qoxAB*	37.4	1.11	6.14 ± 0.01	1.622 ± 0.004	89.8	0.46	5.12 ± 0	0.82 ± 0.02
Δ*cydAB* Δ*qoxAB*	64.8	0.64	5.27 ± 0.01	1.107 ± 0.005	92.2	0.45	5.18 ± 0.06	0.82 ± 0.03

a Td, doubling time.

The final pH of the growth media of the aerobically grown Δ*cydAB* mutant and the Δ*cydAB ΔqoxAB* double mutant was more acidic than that of either the wild type or the Δ*qoxAB* mutant and typical of that seen in anaerobically grown cultures (Table 1). This suggests that even in the presence of oxygen, the Δ*cydAB* mutant grows fermentatively. However, the levels of ethanol accumulated in the growth media of all four strains following aerobic growth were the same, between 0.0014 and 0.0015 g liter^−1^, and in contrast to anaerobic growth, where all four strains accumulated ethanol to between 0.11 and 0.15 g liter^−1^. Acetoin is known to accumulate during aerobic growth of L. monocytogenes (14), and therefore, we measured the levels of acetoin produced during aerobic and anaerobic growth. As predicted, the wild-type strain accumulated acetoin in the medium following overnight aerobic growth (3.60 ± 0.13 mM [standard error {SE}]), as did the Δ*qoxAB* mutant (3.54 ± 0.01 mM). However, both the Δ*cydAB* mutant (0.08 ± 0.02 mM) and the Δ*cydAB ΔqoxAB* double mutant (0.02 ± 0.006 mM) produced very low levels of detectable acetoin following aerobic growth, an outcome more typical of fermentation. Following anaerobic growth, as predicted, all four strains accumulated low levels of acetoin: wild type, 0.091 ± 0.023 mM; Δ*cydAB* mutant, 0.1 ± 0.005 mM; Δ*qoxAB* mutant, 0.121 ± 0.04 mM; and Δ*cydAB ΔqoxAB* double mutant, 0.068 ± 0.009 mM. Collectively, these data indicate that, under aerobic conditions, the Δ*cydAB* mutation abolishes the ability of the strain to use oxygen as the terminal electron acceptor and to undertake respiration. Rather, it undergoes fermentation, but not that typical of anaerobically grown L. monocytogenes, which is likely to be due to many of the genes/enzymes required for fermentation normally not being expressed or being inactive under aerobic conditions (15). In addition, these data indicate that in air the menaquinol *aa*_3_-type oxidase appears not to be capable of compensating for the loss of the cytochrome *bd*-type oxidase, even though spectral analysis showed it is present under these conditions (Fig. 1).

**FIG 2:**
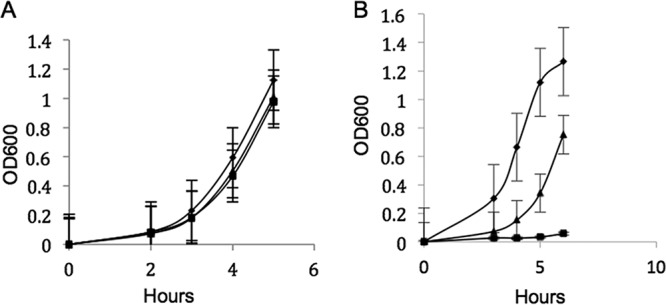
Growth (optical density at 600 nm [OD_600_]) *in vitro* of the wild type (diamonds) and Δ*cydAB* (squares) and Δ*qoxAB* (triangles) mutants in TSB at 37°C in 1% (vol/vol) oxygen (A) or 0.2% (vol/vol) oxygen (B). The data are the means of the results of at least 3 independent experiments ± SE.

It has been reported that in other bacteria the presence of more than one terminal oxidase is important in allowing adaptation to differing levels of oxygen (18–22). To test this for L. monocytogenes, we grew the wild type and the Δ*cydAB* and Δ*qoxAB* mutants with different levels of oxygen provided. At 1% (vol/vol) oxygen, neither the Δ*cydAB* mutant nor the Δ*qoxAB* mutant had a detectable phenotype, and both grew similarly to the wild type (Fig. 2A) and generated similar levels of acetoin (data not shown). These data suggest that at 1% (vol/vol) oxygen, both the menaquinol *aa*_3_-type and the cytochrome *bd*-type oxidases are capable of functioning and the presence of either oxidase is sufficient for aerobic respiration. At 0.2% (vol/vol) oxygen, both mutants had detectable growth phenotypes (Fig. 2B). The Δ*cydAB* mutant barely grew after 6 h, while the Δ*qoxAB* mutant had a pronounced lag phase compared to the wild type (Fig. 2B). These data suggest that at very low levels of oxygen both oxidases are necessary for maximum growth.

### The cytochrome *bd*, but not the menaquinol *aa*_3_, oxidase confers resistance to reactive nitrogen species.

It has been reported that in Escherichia coli and Azotobacter vinelandii, cytochrome *bd* plays a role in resistance to hydrogen peroxide and increased stationary-phase survival (21, 24). We found no difference in the sensitivities of the wild type and the Δ*cydAB*, Δ*qoxAB*, or Δ*cydAB ΔqoxAB* mutant to 3 mM hydrogen peroxide, a concentration above which the growth of the wild type was affected (data not shown), indicating that neither oxidase is involved in resistance to hydrogen peroxide in L. monocytogenes. Likewise, we could not detect any difference in the viability of the wild type or the Δ*cydAB*, Δ*qoxAB*, or Δ*cydAB ΔqoxAB* mutant up to 96 h post-stationary phase (data not shown), indicating that neither oxidase contributes to long-term post-stationary-phase survival.

**FIG 3:**
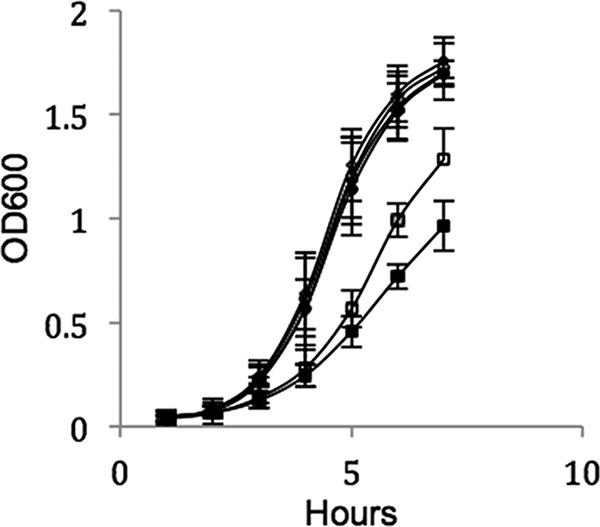
Growth (OD_600_) in TSB of the wild type with (filled diamonds) or without (open diamonds) 10 mM ASN, the Δ*cydAB* mutant with (filled squares) or without (open squares) 10 mM ASN, and the Δ*qoxAB* mutant with (filled circles) or without (unfilled circles) 10 mM ASN. The data are the means of the results of at least 3 independent experiments ± SE.

In Salmonella enterica serovar Typhimurium, the expression of *bd* oxidase has been shown to be important for resistance to the inhibitory properties of nitric oxide (NO) (25), as demonstrated earlier for E. coli (26). To test if this is also the case in L. monocytogenes, the wild type, together with the Δ*cydAB* and Δ*qoxAB* mutants, was exposed to 10 mM acidified sodium nitrite (ASN) as a source of NO (27). The wild type and Δ*qoxAB* mutant were unaffected by the presence of 10 mM ASN, suggesting that the menaquinol *aa*_3_ oxidase is not crucial in conferring resistance to NO (Fig. 3). In contrast, the growth of the Δ*cydAB* mutant was inhibited by the presence of 10 mM ASN (Fig. 3), indicating a role for cytochrome *bd* oxidase in conferring resistance to NO.

### The cytochrome *bd* oxidase, but not the menaquinol *aa*_3_ oxidase, is required for the intracellular growth of L. monocytogenes in air, while either can support intracellular growth in 1% (vol/vol) oxygen.

**FIG 4:**
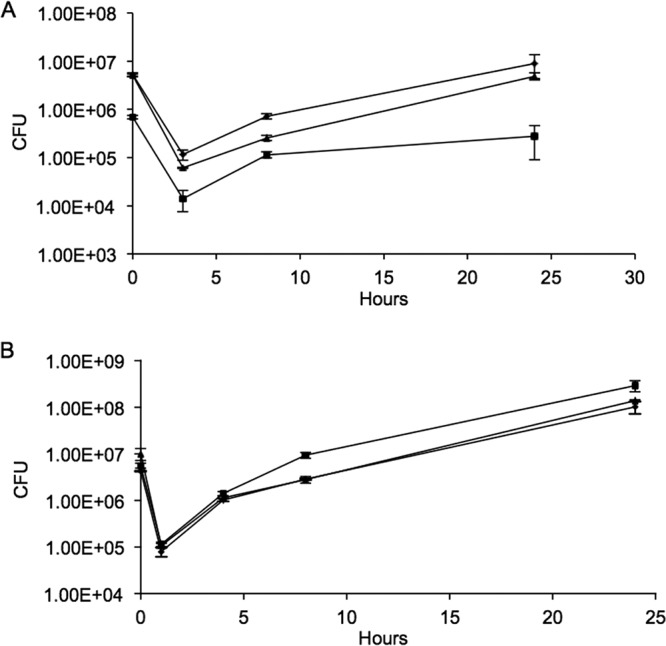
Comparison of the intracellular growth of Δ*cydAB* and Δ*qoxAB* mutants in epithelial cells. HeLa cells were infected at an MOI of 10 with either the wild type (diamonds) or the Δ*cydAB* (squares) or Δ*qoxAB* (triangles) mutant and grown in air (A) or 1% (vol/vol) oxygen (B). Bacterial growth was assessed by lysing the HeLa cells at intervals, followed by serial dilution and counting of viable bacteria. The data are the means of the results of at least six independent experiments ± SE.

**FIG 5:**
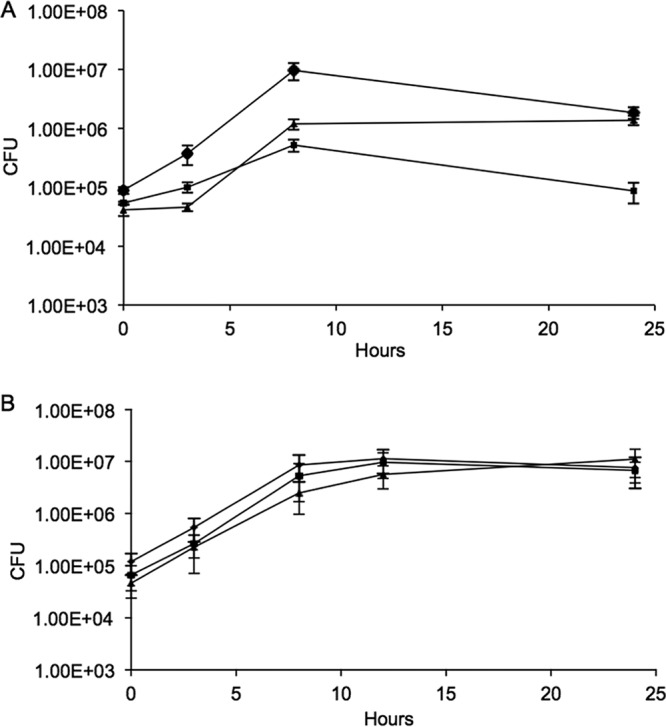
Comparison of the intracellular growth of Δ*cydAB* and Δ*qoxAB* mutants in macrophages. J774 cells were infected at an MOI of 0.5 with either the wild type (diamonds) or the Δ*cydAB* (squares) or Δ*qoxAB* (triangles) mutant and grown in either 20% (vol/vol) oxygen (A) or 1% (vol/vol) oxygen (B). Bacterial growth was assessed by lysing the J774 cells at intervals, followed by serial dilution and counting of viable bacteria. The data are the means of the results of at least five independent experiments ± SE.

It had been reported previously that intracellular growth of L. monocytogenes was predominantly via aerobic metabolism (16). To ascertain the contribution of the cytochrome *bd* and the menaquinol *aa*_3_ oxidases in this process, HeLa cells were infected with the wild type or the Δ*cydAB* or Δ*qoxAB* mutant, and bacterial growth was monitored at intervals over the course of 24 h postinfection. The growth of the Δ*cydAB* mutant in HeLa cells was attenuated, with no increase in intracellular numbers after 10 h (Fig. 4A) and with significantly fewer bacteria than the wild type at 24 h (*P* < 0.001; *n* = 6). The growth defect could be complemented by the presence of the cloned *cydAB* genes on plasmid pCydAB (see Fig. S1 in the supplemental material). In contrast, the growth of the Δ*qoxAB* mutant in HeLa cells was unaffected (Fig. 4A). The growth of the Δ*cydAB* mutant in macrophage-like J774 cells was also attenuated (Fig. 5A), with significantly fewer bacteria than the wild type at 10 and 24 h postinfection (*P* < 0.001; *n* = 5), while the intracellular growth of the Δ*qoxAB* mutant appeared unaffected in these cells (Fig. 5A). The growth defect could be complemented by the presence of the cloned *cydAB* genes on plasmid pCydAB (see Fig. S2 in the supplemental material). These experiments were performed in air and confirm that under such conditions, the lack of cytochrome *bd* oxidase compromises the ability of L. monocytogenes to grow intracellularly and that the menaquinol oxidase *aa*_3_ cannot substitute under these conditions.

To further explore the roles of the two oxidases in intracellular growth, the *in vitro* infections were repeated in 1% (vol/vol) oxygen, where either oxidase alone is proficient for growth (Fig. 2). Under these conditions, neither mutant was compromised in its ability to replicate intracellularly in either HeLa (Fig. 4B) or J774 (Fig. 5B) cells. The double mutant failed to replicate, indicating that under these conditions a functional oxidase and an ability to use oxygen as a terminal electron acceptor were important for intracellular growth (data not shown). The poor survival of both the HeLa and J774 cells at oxygen tensions below 1% prevented further *in vitro* infection assays at an oxygen tension (data not shown).

### Both the cytochrome *bd* and menaquinol *aa*_3_ oxidases are important during murine infection.

**FIG 6:**
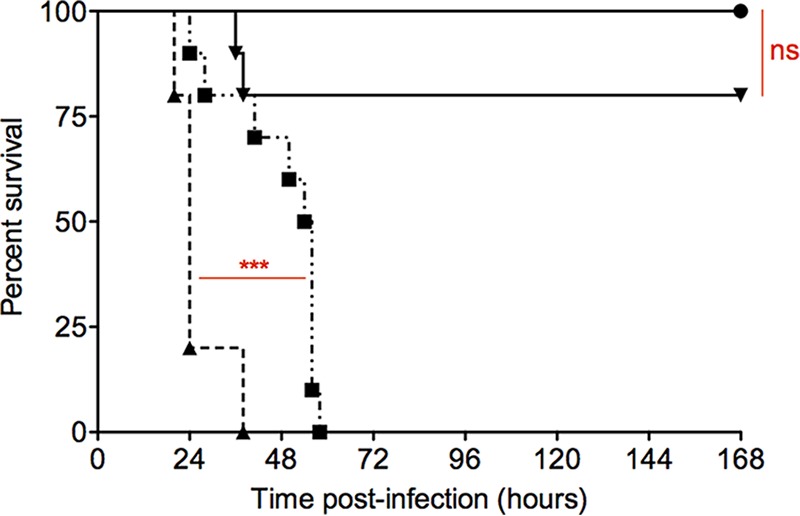
Percent survival of mice following intragastric infection. Mice were infected as described in Materials and Methods with 5.0 × 10^9^ CFU of either the wild type (triangles) or the Δ*cydAB* (inverted triangles), Δ*qoxAB* (squares), or Δ*cydAB ΔqoxAB* (circles) mutant. For each bacterial strain, 10 mice were infected. The experiment was ended after 168 h. Significant differences in survival rates were observed between the wild type and the Δ*qoxAB* mutant (*P* < 0.001) and compared with either the Δ*cydAB* mutant (*P* < 0.001) or the Δ*cydAB ΔqoxAB* mutant (*P* < 0.001). There were no significant differences (ns) in survival rates between the Δ*cydAB* and Δ*cydAB ΔqoxAB* mutants (*P* > 0.05). The data were analyzed with a log rank test and a Gehan-Breslow-Wilcoxon test.

To establish the roles of the cytochrome *bd* and menaquinol *aa*_3_ oxidases during *in vivo* infection, mice were infected by oral gavage, and their survival postinfection was recorded. The Δ*qoxAB* mutant was attenuated, with a significant increase in the mean survival time of the mice from 26 h postinfection for the wild type to 48 h postinfection (*P* < 0.001) (Fig. 6). The mean survival times for the Δ*cydAB* and the Δ*cydAB ΔqoxAB* mutants were 142 h postinfection and 168 h postinfection, respectively; both were significantly different from those of the wild type and the Δ*qoxAB* mutant (*P* < 0.0001) (Fig. 6). The survival times of the Δ*cydAB* and the Δ*cydAB ΔqoxAB* mutants were not significantly different (*P* > 0.05). Analysis of the cumulative average disease score during the course of the infection showed that, after infection with the Δ*qoxAB* mutant, the development of disease signs was delayed compared to infection with the wild type, correlating with the increased survival time (see Fig. S3 in the supplemental material). At the time of death, there were significantly fewer L. monocytogenes bacteria in the livers and spleens of mice infected with either the Δ*cydAB* or the Δ*cydAB ΔqoxAB* mutant than when given the wild type or the Δ*qoxAB* mutant (see Fig. S4 and S5 in the supplemental material). In contrast, there was no significant difference in the bacterial load in the livers and spleens at the time of death after infection with the wild type and the Δ*qoxAB* mutant (see Fig. S4 and S5). These data show that, although to differing extents, both cytochrome *bd* and menaquinol oxidase *aa*_3_ are required for virulence in the murine oral infection model.

## DISCUSSION

In this study, we established that L. monocytogenes has two terminal oxidases, a cytochrome *bd* type (CydAB) and a menaquinol *aa*_3_ type (QoxAB). Spectral analyses (Fig. 1) confirmed the presence of cytochrome *bd* in the wild-type and Δ*qoxAB* strains and its absence in the Δ*cydAB* mutant. In the latter, although the expression levels were low, we obtained evidence for an alternative oxidase with features of a menaquinol *aa*_3_ type, which is consistent with the genomic analysis. The spectral data thus confirm that both oxidases are expressed at 37°C when grown in air, and the transcription of their corresponding genes was also confirmed by reverse transcription (RT)-PCR (data not shown).

Deletion of the *cydAB* genes resulted in a substantially reduced growth rate at 37°C in air. Furthermore, metabolite analysis showed that the Δ*cydAB* mutant grew fermentatively under these conditions, with only low levels of acetoin being produced and greater acidification of the growth medium (Table 1). The low level of ethanol accumulated in the growth medium of the aerobically grown Δ*cydAB* mutant, comparable to that of the wild type, is consistent with expression of the *adh* gene, encoding alcohol dehydrogenase, being repressed under these aerobic conditions (15).

The growth experiments under defined oxygen tension demonstrate interplay between the two oxidases in L. monocytogenes. The observation that the menaquinol oxidase *aa*_3_ was not able to compensate for the loss of the cytochrome *bd* oxidase and to support aerobic respiration in the Δ*cydAB* mutant in air would argue that the cytochrome *bd* oxidase is essential for aerobic respiration in air and that the menaquinol oxidase *aa*_3_ is nonfunctional under these conditions. The observation that the Δ*qoxAB* mutant had no detectable phenotype under these conditions further supports this notion. Recent studies demonstrating that a Δ*cydAB* mutation had a much more significant effect on reducing the proton motive force (PMF) of aerobically grown L. monocytogenes than a Δ*qoxAB* mutation (28) supports the conclusion that under aerobic conditions, CydAB is the critical oxidase.

The finding that at 1% (vol/vol) oxygen neither the Δ*cydAB* nor the Δ*qoxAB* mutant had a detectable growth phenotype (Fig. 2A) indicates that at this level of oxygen the two oxidases are equally functional. However, at 0.2% (vol/vol) oxygen, growth of both the Δ*cydAB* and Δ*qoxAB* mutants was inhibited, with that of the Δ*cydAB* being most pronounced (Fig. 2B). This indicates that at this low oxygen tension both oxidases are required for maximal growth, with neither oxidase alone being capable of supporting growth comparable to that of the wild type. The poor growth of the Δ*cydAB* mutant in 0.2% (vol/vol) oxygen compared to its growth anaerobically probably reflects a combination of a lack of a functional *bd* oxidase under low-oxygen conditions and inhibition by the presence of oxygen of the expression of genes that are expressed under anaerobic conditions. However, these data demonstrate that cytochrome *bd* oxidase functions across a broad range of oxygen tensions, while menaquinol *aa*_3_ oxidase functions only at low oxygen tensions.

The role of the cytochrome *bd* oxidase in L. monocytogenes, therefore, differs from that of its counterpart in E. coli, where cytochrome *bd* oxidase has been shown to have a high affinity for oxygen, allowing growth under microaerobic conditions (29). Likewise, in Klebsiella pneumoniae, cytochrome *bd* oxidase is important in removing low-level oxygen in order to protect oxygen-sensitive reactions, such as nitrogen fixation (18). However, there is a precedent for an essential role for cytochrome *bd* at higher oxygen levels: in A. vinelandii, mutation of cytochrome *bd* showed that it is required for protection of nitrogenase from oxygen damage at ambient oxygen levels (30, 31). This is thought to be consistent with the exceptionally high turnover rates of the oxidase in A. vinelandii and perhaps the lower affinity for oxygen (32).

The *in vitro* infection assays demonstrate the importance of aerobic respiration for the growth of L. monocytogenes in both epithelial cells and macrophages (Fig. 4A and 5A). This confirms earlier findings that showed that the intracellular growth of L. monocytogenes aro mutants, which are incapable of synthesizing menaquinone, is highly attenuated (16). The importance of *bd* oxidases for intracellular growth has been shown in a number of pathogens. In Shigellla flexneri, *bd* oxidase was required for intracellular survival and virulence (33), and in Mycobacterium tuberculosis, which has both a *bd* and an *aa*_3_ oxidase, it has been shown that in moving from acute to chronic infection there is a shift in expression to *bd*-mediated respiration, with mutants defective for *bd* oxidase being unable to establish persistent lung infections (34). The *in vitro* infection experiments performed at 1% (vol/vol) oxygen showed that the Δ*cydAB* and Δ*qoxAB* mutants grew similarly to wild-type bacteria in both epithelial cells and macrophages (Fig. 4B and 5B). This is consistent with the growth experiments, where both the Δ*cydAB* and Δ*qoxAB* mutants grew as well as the wild type in 1% (vol/vol) oxygen (Fig. 2A), and confirms that at this level of oxygen, both oxidases are functional and either can support aerobic respiration and permit effective intracellular replication. The apparent discrepancy with the conclusion of recent studies that intracellular growth of a Δ*cydAB* mutant is unaffected in macrophages (28) can be explained by the shorter time frame of 8 h used in those experiments (28). The attenuation of the intracellular growth of the Δ*cydAB* mutant in macrophages was less pronounced at 8 h in our study but much clearer at a later time point (Fig. 5A). Our observations here are in keeping with an important role for CydAB oxidase-mediated respiration during intracellular growth and are consistent with the reported reduction in plaque-forming ability of the Δ*cydAB* mutant (28). Taken as a whole, these data show a clear requirement for aerobic respiration for intracellular growth and that, depending on the oxygen conditions, either oxidase in L. monocytogenes can function in this capacity. The increased sensitivity of the Δ*cydAB* mutant to reactive nitrogen species in the form of ASN (Fig. 3) indicates an additional role for cytochrome *bd* oxidase, namely, as part of the anti-reactive-nitrogen response in L. monocytogenes. It has been shown that in both *S*. Typhimurium and uropathogenic E. coli (UPEC) the expression of a *bd* oxidase is the main contributor to NO tolerance and host colonization (25, 35). The interplay between L. monocytogenes and the production of NO by the host is more complex. While it is known that Nos2^−/−^ mice are more susceptible to infection with L. monocytogenes (36), it has also been shown that the presence of NO both reduces survival of cells infected with L. monocytogenes and promotes the spread of L. monocytogenes into uninfected cells (37, 38). Thus, it appears that L. monocytogenes may exploit the production of NO by the host to promote its survival and spread. As such, an ability to resist the action of NO via cytochrome *bd* could be important in allowing L. monocytogenes to resist the killing effects of NO and thereby promote its own survival in infected cells.

The murine infection data show clear roles for both oxidases in infection. The Δ*cydAB* and Δ*cydAB ΔqoxAB* mutants are both highly attenuated (Fig. 6). The Δ*qoxAB* mutant is also attenuated, with a significant increase in survival time (48 h postinfection) of infected mice compared to mice infected with the wild-type strain (26 h postinfection) (*P* < 0.001). This clearly shows that both oxidases are required for infection but that cytochrome *bd* oxidase is most critical for maximum virulence. This is in keeping with the *in vitro* infection data that showed the Δ*cydAB* mutation had the most dramatic effect on intracellular replication in epithelial cells and macrophages. The significant reduction in the numbers of L. monocytogenes bacteria in the livers and, most dramatically, the spleens of mice infected with the Δ*cydAB* and Δ*cydAB ΔqoxAB* mutants at the time of death shows that functional cytochrome *bd* is critical during this stage of *in vivo* infection (see Fig. S4 and S5 in the supplemental material). Furthermore, these data indicate that aerobic respiration is critical for growth in hepatocytes and cells in the spleen. While the Δ*qoxAB* mutant had no detectable phenotype during *in vitro* intracellular growth, analysis of the cumulative average disease scores of mice infected with the Δ*qoxAB* mutant (see Fig. S3 in the supplemental material) demonstrated that the mutant is delayed in the development of the infection, pointing to a role for the menaquinol *aa*_3_ oxidase in the initial stages following oral infection. It is known that in the lumen of the small intestine and at the villus tip of the mucosal surface, the levels of oxygen are low, routinely <1.0% (39). Therefore, our interpretation of the delay in the onset of disease signs in infections with the Δ*qoxAB* mutant is that, following oral gavage, the Δ*qoxAB* mutant experiences a low-oxygen environment (less than 1%), which impacts its growth rate and results in a delay in invasion. However, in subsequent stages of infection, menaquinol *aa*_3_ oxidase is dispensable for growth, as demonstrated by equal numbers of the mutant and wild-type bacteria in the livers and spleens of mice at the time of death (see Fig. S4 and S5 in the supplemental material). With Staphyloccus aureus, it has been demonstrated that the Cyd and Qox oxidases are both required for successful infection in a murine model, with loss of either oxidase leading to defects in organ-specific colonization (40). Interestingly in that case, the *cydB* mutant was capable of colonization of the liver but not the heart, whereas the opposite was true for the *qoxB* mutant (40). Clearly, the data presented here add to the growing number of reports that the ability of pathogens to tailor their respiratory mechanisms to the microenvironments encountered in the host, and in responding to the host innate immune response, can be critical to the successful establishment of infection.

It has been reported previously that aerobic respiration in L. monocytogenes requires the response regulator ResD, which activates transcription of the *cydAB* genes (17). In these experiments, a *resD* mutant showed reduced growth in rich medium, but with a less pronounced growth defect compared to the wild type than was seen in the phenotype of the Δ*cydAB* mutant described in our study. In addition, the *resD* mutation did not affect intracellular replication in either HeLa or J774 cells or in a mouse model of infection (17). This lack of a phenotype for a *resD* mutant seems curious and is at odds with the findings reported here for the Δ*cydAB* mutant and the previously described findings for *aro* mutants, which are also unable to undergo aerobic respiration (16). We believe this difference can be explained by the fact that the *resD* mutation did not completely abolish transcription of the *cydAB* genes (17). As such, the less dramatic phenotype of a *resD* mutant may be explained in part by continuing low-level expression of the *cydAB* genes.

In conclusion, we have demonstrated that L. monocytogenes has two terminal oxidases, a cytochrome *bd* type (CydAB) and a menaquinol cytochrome *aa*_3_ type (QoxAB), both of which are important for different stages of infection. These findings are consistent with the ability of L. monocytogenes to switch between different terminal electron acceptors under varying oxygen concentrations, allowing adaptation to different conditions encountered within the infected host.

## MATERIALS AND METHODS

### Bacterial strains and culture conditions.

L. monocytogenes serotype 1/2a strain EGDe:InlA^m^, engineered for murine oral infection (41), was used as the wild type, and all mutations were generated in this background. The InlA^m^ mutation has no effect on the ability of the strain to infect human cells *in vitro* (42, 43). L. monocytogenes was cultured in TSB (Oxoid), which was supplemented with 5 μg ml^−1^ erythromycin when plasmids were maintained. Cultures were grown anaerobically using 10-ml Luer-locked syringes completely filled with degassed TSB. Growth and *in vitro* infections in known concentrations of oxygen (0.1% to 5% [vol/vol] O_2_, 5% [vol/vol] CO_2_; 37°C) were performed using a humidified hypoxic chamber (Coy Laboratory Products, United Kingdom), as described previously (44). Acetoin production was measured using the Voges-Proskauer test as described previously (14). NO was generated as described previously (27). Ethanol production was measured using an ethanol assay kit (Megazyme, Ireland).

### Spectral analysis of cytochromes.

Difference spectra (i.e., the difference between the spectrum of a reduced sample minus the spectrum of an oxidized sample or, alternatively, the difference between a reduced plus CO sample minus a reduced sample) of suspensions of intact cells suspended in buffer (10 mM Tris-HCl, pH 7.0) were recorded in a dual-wavelength spectrophotometer using a 10-mm-path-length cuvette. The cells were reduced by the addition of a few grains of dithionite and then treated with CO bubbling from a cylinder, the gas from which was sparged through alkaline pyrogallol to deplete oxygen. Spectra were recorded in triplicate. Alternatively, samples were oxidized with a few grains of ammonium persulfate. Protein concentrations were measured using the Markwell assay (45).

Electronic absorption spectra were recorded using a custom-built SDB4 dual-wavelength scanning spectrophotometer (University of Pennsylvania School of Medicine) detailed previously (46). The instrument was based on earlier dual-wavelength configurations (47). Light from a 45-W tungsten halogen source was focused on the entrance slits of two Jobin-Yvon H20 monochromators, one of which was driven by a J-Y TTL stepper interface over the desired spectral range, while the second was set at the reference wavelength. The output of the monochromators was modulated by a resonant tuning fork vibrating mirror and focused on the cuvette. Transmitted light was measured with a Hamamatsu R928 side window photomultiplier tube positioned about 50 mm from the nearest edge of the cuvette. Spectra were generally scanned at approximately 4.25 nm s^−1^ at 10 samples per point with a 0.5-nm step size. The slit width was 0.5 mm, giving a spectral bandpass of 2 nm. Unsmoothed or smoothed spectral data were analyzed and plotted using SoftSDB (Current Designs) and Excel software.

### Molecular cloning and generation of L. monocytogenes mutants.

Gene cloning and PCR were performed using standard methods. The Δ*cydAB* and Δ*qoxAB* mutants were generated using the temperature-sensitive shuttle plasmid pAUL-A (23). After transformation into L. monocytogenes, induced integration of the plasmid at 42°C, and the second recombination event, only the translational start and stop codons and fewer than 8 codons of each open reading frame remained. Successful generation of mutants was confirmed by colony PCR and subsequent nucleotide sequence determination. Complementation of each mutant was achieved by expression of the cloned wild-type gene on plasmid pUNK1 as described previously (48).

### Cell culture and infection.

HeLa cells (ATCC CCL-2) or J774 macrophages were maintained in Dulbecco's modified Eagle medium (DMEM) (Sigma) containing 10% (vol/vol) fetal bovine serum (FBS) (Sigma) and incubated at 37°C in humidified air with 5% (vol/vol) CO_2_. For infection with L. monocytogenes, semiconfluent cell monolayers were washed twice with prewarmed phosphate-buffered saline (PBS) before infection with mid-log-phase L. monocytogenes in 1 ml serum-free medium at a multiplicity of infection (MOI) of 10:1 for HeLa cells or 0.5:1 for J774 cells for 2 h. After three washes, the cells were incubated in DMEM with 10 μg gentamicin ml^−1^ for the wild type and Δ*qoxAB* mutant and 25 μg ml^−1^ for infections using Δ*cydAB* mutants until the desired time points postinfection were reached, when the cells were lysed with 0.5% (vol/vol) Triton X-100 in PBS and the total viable count was determined. Statistical analyses were performed using Student's *t* test, where a *P* value of <0.05 indicated a statistically significant difference.

### Virulence of L. monocytogenes strains in mice.

Female CD1 outbred mice (Charles River, United Kingdom) were purchased at 8 weeks and housed for a week to acclimatize before infection. Oral gavage was performed as described previously (41), except that bacterial suspensions were inoculated intragastrically into mice that had not been starved and inoculation was performed using a 21G soft cannula attached to a 1-ml syringe and a dose of 5 × 10^9^ CFU in 500 μl of PBS. Blood was collected at 24 h postinfection to determine the CFU numbers. The mice were monitored periodically for signs of disease, based on Morton's scheme (49), and when they reached a severely lethargic state, they were terminally anesthetized to collect blood by cardiac puncture to determine the CFU in the blood at the time of death [ToD]. Animals were culled immediately by cervical dislocation, and the ToD was recorded. Mice alive at 7 days (168 h) postinfection were deemed to have survived the infection. Postmortem tissue collection of liver and spleen was performed to determine the CFU in the tissue. Statistical analysis was performed using the Gehan-Breslow-Wilcoxon test.

## Supplementary Material

Supplemental material
