# Supplementary material for: Listeria monocytogenes Has Both Cytochrome bd-Type and Cytochrome aa3-Type Terminal Oxidases, Which Allow Growth at Different Oxygen Levels, and Both Are Important in Infection
Source: Infect Immun. 2017 Oct 18;85(11):e00354-17. doi: 10.1128/IAI.00354-17 (PMC5649020; doi:10.1128/IAI.00354-17)
Supplement: Supplemental material [file supp_85_11_e00354-17__index.html]

Supplemental material 

# Listeria monocytogenes Has Both Cytochrome *bd*-Type and Cytochrome *aa*3-Type Terminal Oxidases, Which Allow Growth at Different Oxygen Levels, and Both Are Important in Infection

## Supplemental material

- Supplemental file 1 -

  Fig. S1. Intracellular growth in epithelial cells of the complemented Δ*cydAB* mutant. Fig. S2. Intracellular growth in macrophages of the complemented Δ*cydAB* mutant. Fig. S3. Cumulative average disease score in mice intragastrically infected with *L. monocytogenes* strains over a 168-hour time course. Fig. S4. Number of bacteria per mg of liver tissue at the endpoint of the infection. Fig. S5. Number of bacteria per mg of spleen tissue at the endpoint of the infection.

  PDF, 178K
