# Supplementary material for: Listeria monocytogenes Has Both Cytochrome bd-Type and Cytochrome aa3-Type Terminal Oxidases, Which Allow Growth at Different Oxygen Levels, and Both Are Important in Infection
Source: Infect Immun. 2017 Oct 18;85(11):e00354-17. doi: 10.1128/IAI.00354-17 (PMC5649020; doi:10.1128/IAI.00354-17)
Supplement: Supplemental material [file IAI.00354-17_zii999092194s1.pdf]

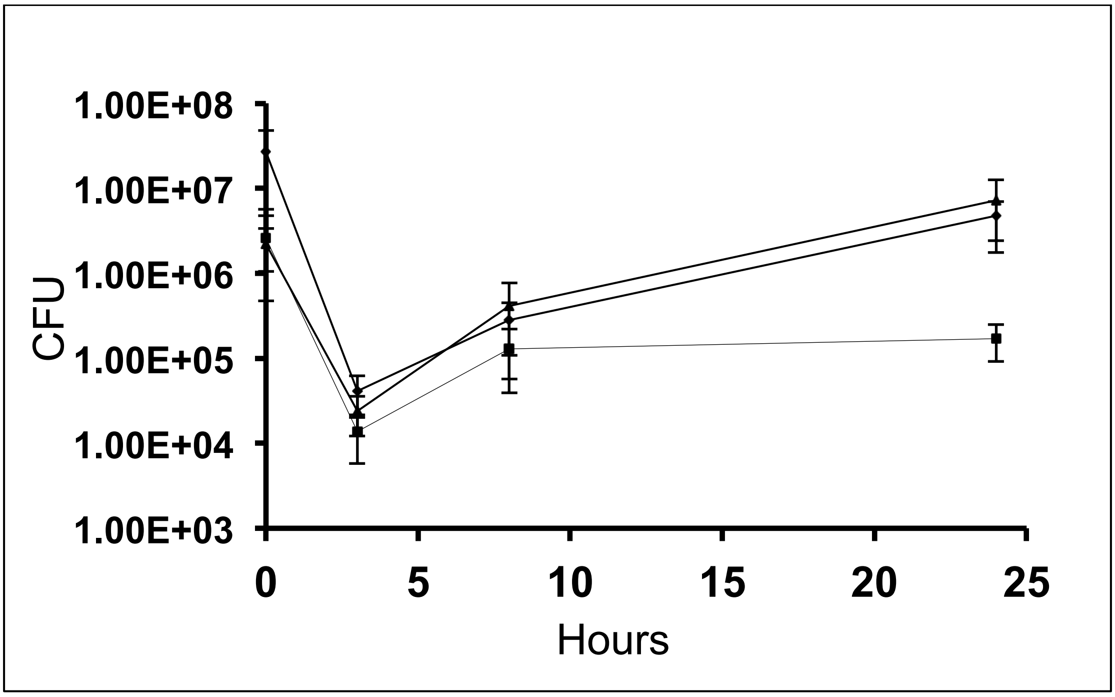

FIGURE S1.

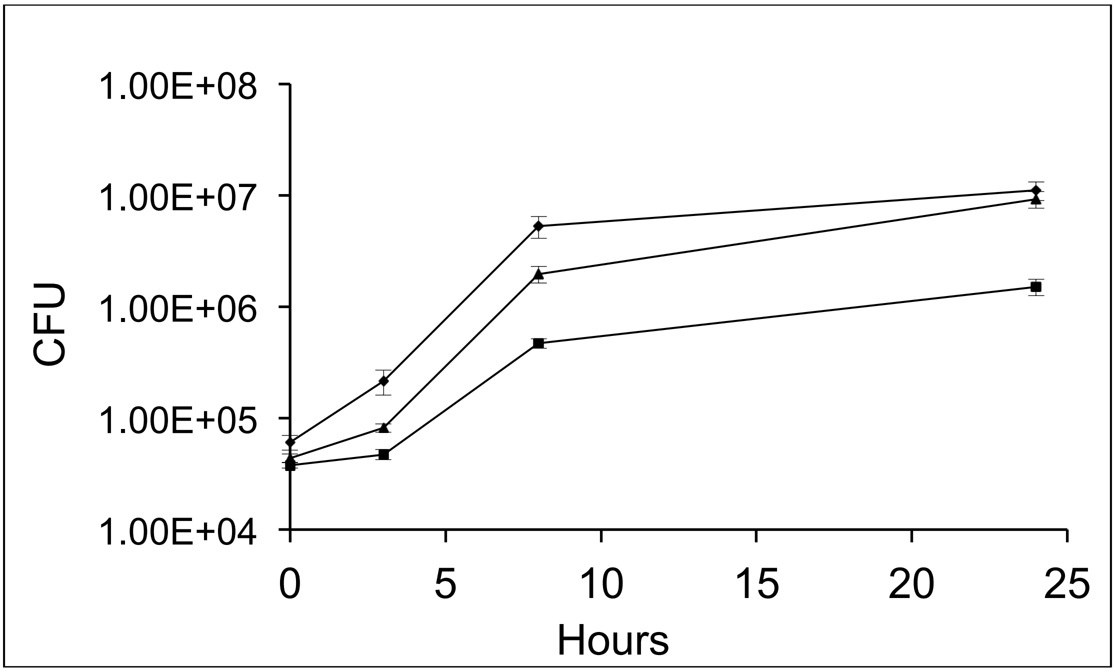

FIGURE S2.

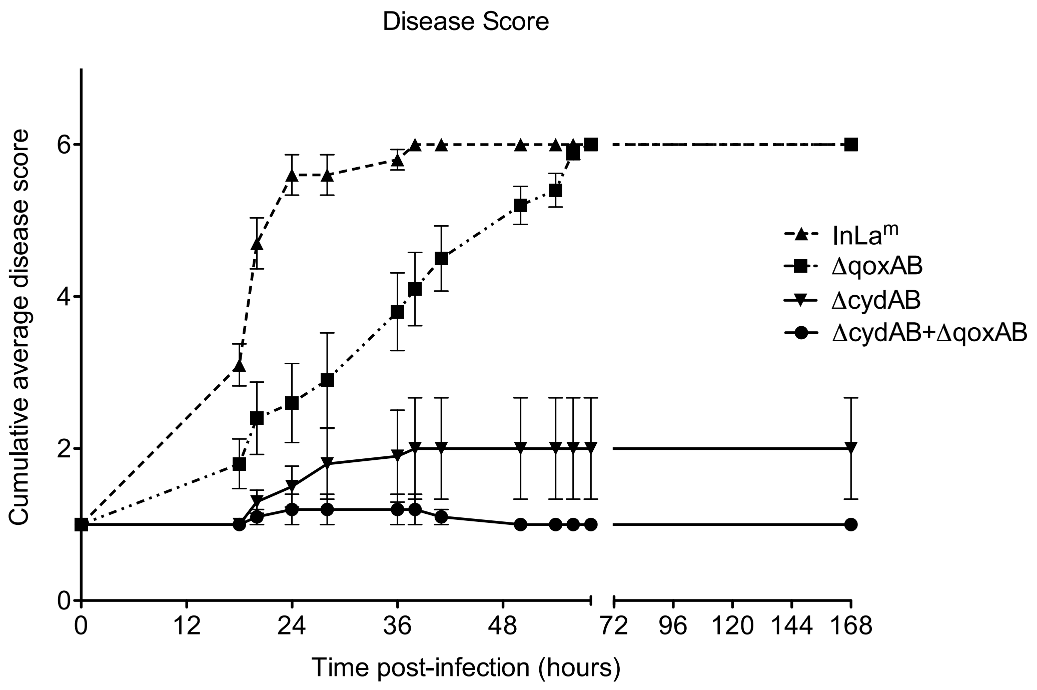

Stats:
InLa^m *vs* ΔqoxAB: $p < 0.001$
ΔqoxAB *vs* ΔcydAB: $p < 0.0001$
ΔcydAB *vs* ΔcydAB+ΔqoxAB: $p > 0.05$

Average survival time:
InLa^m: 26 hours p.i.
ΔqoxAB: 48 hours p.i.
ΔcydAB: 142 hours p.i.
ΔcydAB+ΔqoxAB: 168 hours p.i.

FIGURE S3

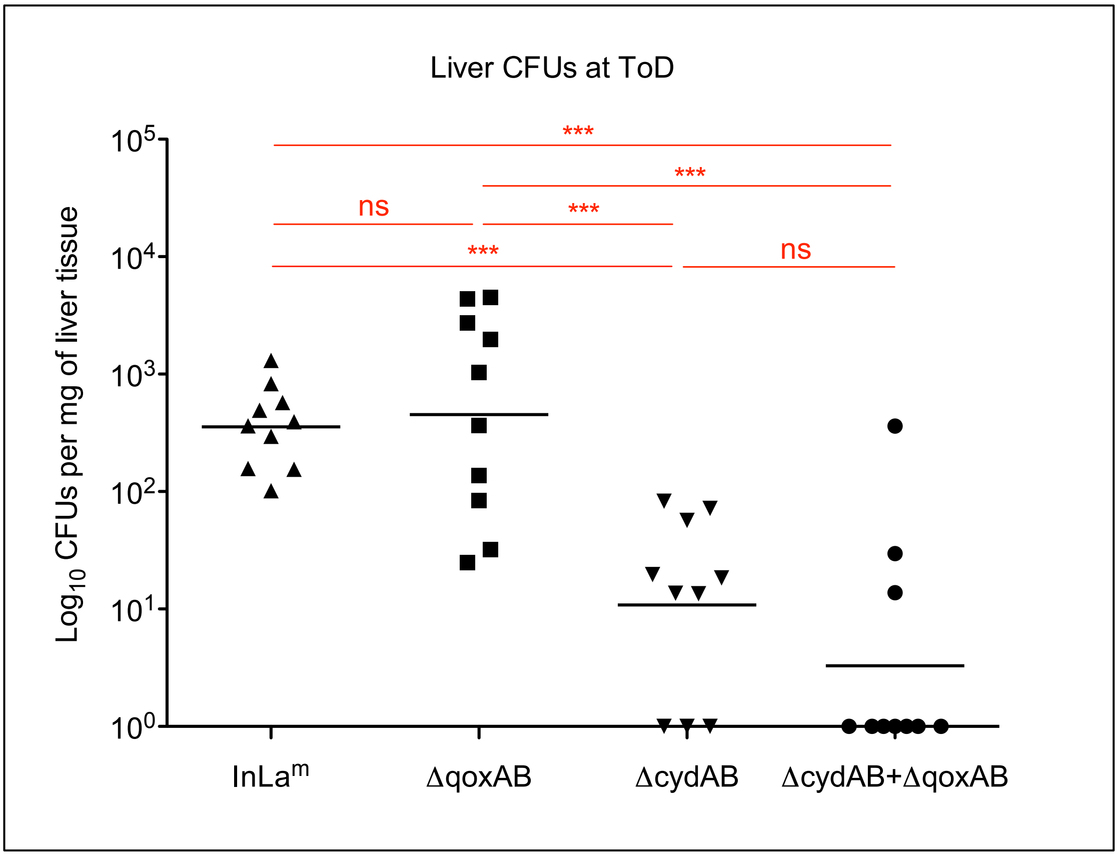

FIGURE S4

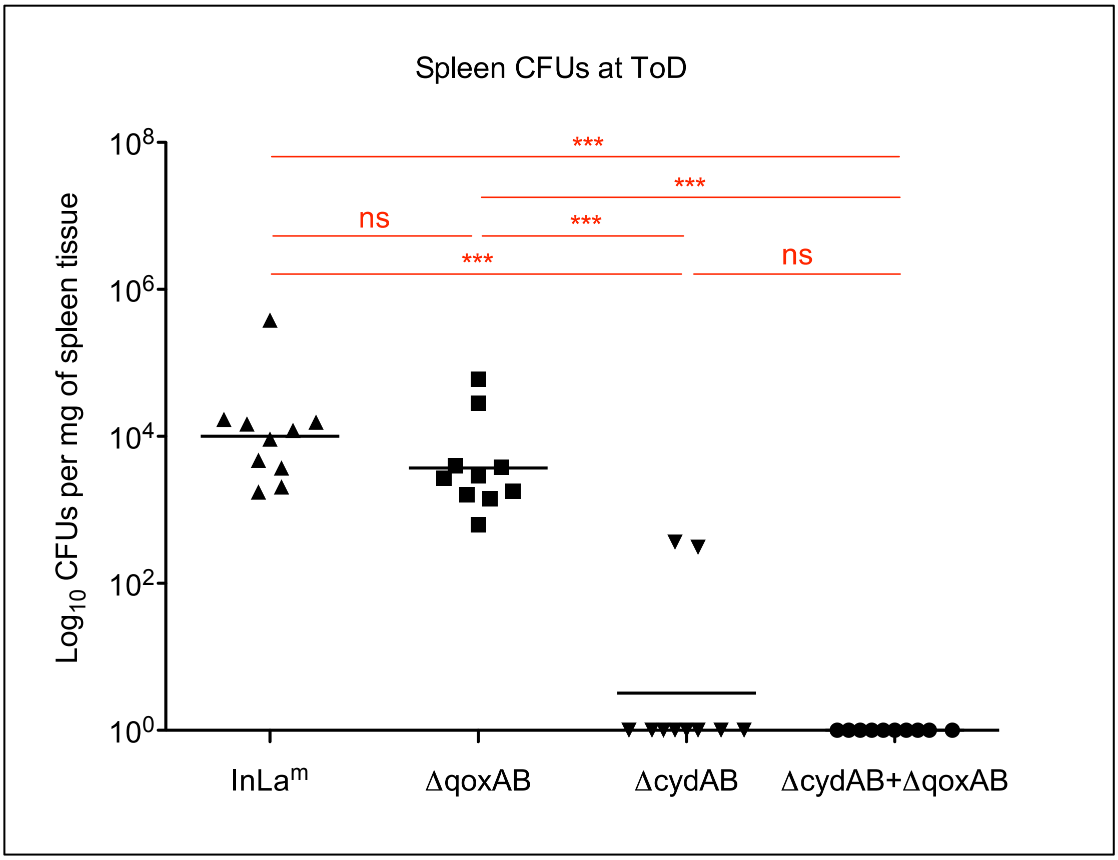

Figure S5

Supplementary Figure Legends.

Figure S1.

The intracellular growth in epithelial cells of the complemented *ΔcydAB* mutant. HeLa cells were infected at an MOI of 10 with either the wild type (triangles), *ΔcydAB* (squares) or *Δ*cydAB(pCydAB) (diamonds) and grown in air. Bacterial growth was assessed by lysing HeLa cells at intervals followed by serial dilution and viable counting of bacteria. Data are the means of at least three independent experiments ± S.E

Figure S2.

The intracellular growth in macrophages of the complemented *ΔcydAB* mutant. J774 cells were infected at an MOI of 0.5 with either the wild type (diamonds), *ΔcydAB* (squares) or *ΔcydAB*(pCydAB) (triangles) and grown in air. Bacterial growth was assessed by lysing J774 cells at intervals followed by serial dilution and viable counting of bacteria. Data are the means of at least three independent experiments ± S.E.

Figure S3.

Cumulative average disease score in mice intragastrically infected with *L. monocytogenes* strains over 168-hour time-course. Each mouse was frequently monitored and scored (1 = normal, 6 = lethargic), based on Morton's scheme (1). The time to the end point was recorded as the "survival time". Conditions were compared with Two-way ANOVA. Error bars represent ± SEM. Statistical significance is denoted with $P \leq 0.05$.

1. **Morton DB, Griffiths, PH.** 1985. Guidelines on the recognition of pain, distress and discomfort in experimental animals and a hypothesis for assessment. *The Veterinary Record*. **116:** 431-436.

Figure S4.

The number of bacteria per mg of liver tissue at the end-point of the infection. Each point refers to one mouse (n = 10 mice per bacterial strain). Conditions were compared with individual *t*-tests. Statistical significance is denoted with $P \leq 0.05$. NS is not significant $P > 0.05$.

Figure S5.

The number of bacteria per mg of spleen tissue at the end-point of the infection. Each point refers to one mouse (n = 10 mice per bacterial strain). Conditions were compared with individual *t*-tests. Statistical significance is denoted with $P \leq 0.05$. NS is not significant $P > 0.05$.
